# Novel molecular subgroups within the context of receptor tyrosine kinase and adhesion signalling in multiple myeloma

**DOI:** 10.1038/s41408-021-00442-2

**Published:** 2021-03-04

**Authors:** Ellen Leich, Martin Schreder, Jordan Pischimarov, Thorsten Stühmer, Torsten Steinbrunn, Martina Rudelius, Daniela Brünnert, Manik Chatterjee, Christian Langer, Sarah Keppler, Sofia Catalina Heredia-Guerrero, Hermann Einsele, Stefan Knop, Ralf Christian Bargou, Andreas Rosenwald

**Affiliations:** 1 Institute of Pathology, University of Würzburg, Würzburg, Germany; 2 Department of Internal Medicine II, University Hospital of Würzburg, Würzburg, Germany; 3 First Department of Medicine, Klinik Ottakring, Vienna, Austria; 4 Comprehensive Cancer Center Mainfranken, University Hospital of Würzburg, Würzburg, Germany; 5 Institute of Pathology, Ludwig-Maximilians-University München, München, Germany; 6 Department of Internal Medicine III, University Hospital Ulm, Ulm, Germany

**Keywords:** Myeloma, Cancer genomics, Cancer therapy, Cancer genetics, Translational research

Dear Editor,

Multiple myeloma (MM) is characterised by a heterogeneous clinical presentation and pronounced differences in survival outcome^1^, reflecting extensive interpatient and intratumour genomic heterogeneity^2^. This might be one of the major reasons why individualised therapeutic concepts that specifically improve the survival of patients with high-risk MM or avoid overtreatment of patients with indolent presentations are generally missing^3^. However, we and others have shown that mutations cluster in signalling networks that are potentially relevant for the course and treatment of this cancer^4–8^. Our previous sequencing study showed that almost 100% of MM cases harbour at least one genetic hit within a small signalling network of adhesion molecules, receptor tyrosine kinases (RTK) and their effectors, suggesting that this network might represent some common ground in MM pathogenesis^6^. About half of the MM cases were even affected by more than one mutation within this network and mutations in RTKs were associated with a worse outcome and the presence of high-risk features such as 17p deletions^9,10^. Here we performed whole-exome sequencing (WES) of primary MM samples to identify molecular subgroups (MSG) within the context of RTK- and adhesion signalling and to understand their association with other recurrent oncogenic events like MYC expression.

Bone marrow aspirates and corresponding peripheral blood mononuclear cells were obtained from 43 MM patients (11 untreated MM and 32 with relapsed/refractory disease) treated at a single institution as part of their routine diagnostic workup after providing informed consent (reference number 18/09). Follow-up biopsies of 18 patients were collected for longitudinal monitoring. Plasma cell purification, WES and Sanger sequencing were performed as previously described^6^ and MYC expression was assessed by immunohistochemistry in all biopsies (thus including biopsies from 20 patients at the time of diagnosis). Visualisation of gene-mutation frequencies and screening of the generated single nucleotide variant (SNV) dataset for mutations in adhesion molecules (*n* = 642), RTKs (*n* = 74), and their effectors (*n* = 63) (Supplementary Table S1) was accomplished with Python-Scripts and integration of aliases/synonyms from the HGNC database. Notably, the scripts allowed to match genes with name analogy. Whether those genes that were not included in the initial list could be assigned to either RTKs, adhesion molecules or effectors was decided upon specific literature research (Supplementary Material and Methods).

Clinical characteristics of the study population are shown in Supplementary Table S2 and Supplementary Results. To assign MM samples to certain molecular categories (mutated in RTKs (RTK^mut^), adhesion molecules, effectors) at least one SNV/mutation had to be technically verified within this category by Sanger sequencing or at least three independent genes of this category had to be affected by a somatic mutation in this specific MM sample (Supplementary Table S3).

From a total of 67 samples (derived from 43 patients), 18, 60 and 49 samples were affected by SNVs in RTKs, adhesion molecules and RTK effectors, respectively (Supplementary Table S4 A–C). Samples from two patients had to be excluded (polyclonal, lack of precise identity) and subsequent correlations were carried out on 63 biopsies from 41 patients (Supplementary Results and Supplementary Fig. S1 A, B). All MM could be assigned to the above-mentioned categories, according to their mutation profile (Fig. 1A, B and Supplementary Table S5 A). Ten biopsies from six patients were exclusively affected by mutations in adhesion molecules and were thus assigned to subgroup MSG1 while the majority (53 biopsies from 31 patients) carried mutations in RTKs and/or their effectors and were assigned to subgroup MSG2 (Fig. 1A and Supplementary Table S5 A). For patients with longitudinal information, all but one were found to remain within their MSG (Fig. 1B and Supplementary Table S5 B), although the global SNV profiles for the different longitudinal samples from any specific patient varied. All samples with classical high-risk features^11–13^ (del17p, *t*(14;16), *t*(14;20) and/or extramedullary disease) at the time of diagnosis and 15/16 samples with such features at the time of biopsy had been assigned to MSG2 (Fig. 1B). Also, MM with high MYC expression (MYC^high^, ≥40% positive plasma cells) were restricted to MSG2 (Fig. 1B–D, *P* < 0.05) and a striking impact of MYC^high^ on overall survival (OS) was observed (Fig. 1E, *P* = 0.028 (39.0 vs 11.1 months)), in confirmation of similar findings^14^. Specifically, 23/35 MM patients (~66%) of MSG2 with MYC-expression data available were MYC^high^ either at all time points (12/28 patients with longitudinal samples) or became so during disease progression (7/28 patients with sequential samples) (Fig. 1F and Supplementary Table S6). Likewise, the integration of RTK^mut^ and MYC^high^—both of which are exclusively associated with MSG2—was strongly associated with high-risk features (*P* < 0.01, Fig. 1G) and worse outcome (*P* = 0.021, Fig. 1H), even though the association with RTK^mut^ or MYC^high^ alone with high-risk features revealed no or only low significance, suggesting that these factors complement each other and seem to be independent adverse features within MSG2. Notably, MM patients that were both MYC^high^ and RTK^mut^ (*n* = 7) had a median OS of only 7.9 months whereas OS has not yet been reached for patients with RTK^WT^ and/or MYC^low^ at the time of this analysis. The number of MM patients with high tumour mutational burden (TMB^high^) was not significantly different between MSG1 and MSG2, but the overall TMB was significantly higher in MSG2, including three patients with TMB of >400 SNVs. Moreover, TP53 mutations (TP53^mut^) were exclusively found in MSG2 (8/43 MM patients; 19%), including five cases with prognostically adverse bi-allelic TP53 lesions^15^ and an OS of only 8.8 months (Supplementary Table S3 and Fig. 1B). Detailed *P* values (patient- and biopsy-specific) are listed in Supplementary Table S7.

**Fig. 1:**
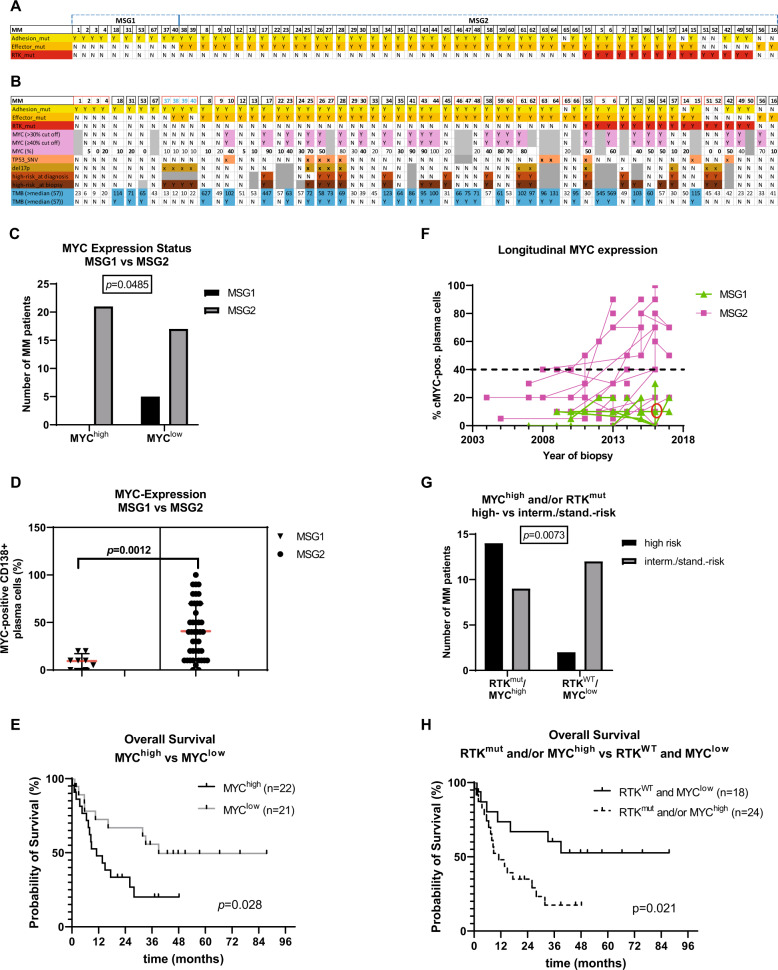
Definition of the two molecular subgroups MSG1 and MSG2 and their correlations with distinct molecular features. Visual overview of the molecular subgroups MSG1 and MSG2 and their correlation with MYC expression, TP53 mutations (TP53_SNV), deletions in 17p (del17p), and overall high-risk status. MM patients (*x* axis) were assigned to the subgroups MSG1 (mutations only in adhesion molecules (yellow)) and MSG2 (mutations in RTKs (red) and RTK effectors (orange)). The separation of the individual patients is indicated by small gaps (**A**). Of the MM patients with longitudinal information available (red rectangle) all but one ((MM37-40), T/N (71/52; 85/86; 99/-; 149/-, in blue)) retained their original subgroup designation (**B**). The frequency of MM with MYC expression ≥40% was higher in MSG2 (*n* = 42, grey bars) than in MSG1 (*n* = 8, black bars) (**C**) and the median MYC expression was significantly lower in MSG1 compared to MSG2 (10% vs 40%) (**D**). Median OS from the time of biopsy was 39.0 months for patients with MYC expression <40% (MYC^low^, *n* = 21, solid line) versus 11.1 months for patients with MYC expression ≥40% (MYC^high^, *n* = 22, dashed line). **E** Longitudinal MYC expression in all sequential trephine biopsies of each MM patient included in the study. The majority of MSG2 MM (pink squares) were either MYC^high^ throughout the disease or were initially MYC^low^ and became MYC^high^ later. MSG1 MM (green triangles) always remained MYC^low^ throughout the disease. Please note that all samples of the patient with biopsies MM37-40 are labelled in green although this patient switched subgroups from MSG1 (MM37) to MSG2 in 2016 (MM38 and 39, red circle) and back to MSG1 (MM40) in 2017. **F** Integration of MYC expression and RTK^mut^ status and subsequent correlation with risk status using Fisher’s exact test (**G**) and OS using the Kaplan Meier method (**H**). Median time of OS from biopsy for cases with RTK^mut^ and/or MYC^high^ (1–2) was 11.1 months, while the median OS for cases with neither RTK^mut^ nor MYC^high^ (0) was not reached yet. MSG: molecular subgroup, RTK: receptor tyrosine kinase, mut: mutation, SNV: single nucleotide variation, yellow: mutation in adhesion molecules, orange: mutation in effector molecules, red: mutation in RTKs, light purple: high MYC expression (≥40%/>30% of plasma cells), salmon: mutation in *TP53*, ochre: del17p, light brown: high-risk at diagnosis, dark brown: high-risk at biopsy, turquoise: TMB > 57 SNVs. Y: yes, N: no, grey: not available.

In contrast, MSG1 MM remained MYC^low^ throughout the observation period (Fig. 1F and Supplementary Table S6) and was not specifically associated with established oncogenic events as seen in MSG2. The clinical course in this small subgroup (*n* = 6) appeared favourable with a median OS > 12 years. This included a patient (samples MM37-40, T/N (71/52; 85/86; 99/-; 149/-)) who presented with del17p at biopsy and was initially assigned to MSG1, switched to MSG2 at a later time-point, but then again maintained an MSG1-MM pattern in subsequent biopsies (Fig. 1B). MYC expression was low (≤20% of plasma cells) in all biopsies acquired from 2007 to 2017 (*n* = 9). In contrast to all other patients with high-risk disease represented within the MSG2 group, this patient experienced a benign course with an OS of 13 years from diagnosis of smouldering MM and 39 months from the start of treatment. The affiliation to MSG1 may thus bestride the presence of high-risk features.

Annotation by STRING network analysis revealed that 241 genes selectively mutated in MSG1 patients were significantly enriched in networks at both low (*P* = 0.024) and highest confidence (*P* = 0.006) (Fig. 2A). The core network that was found at the highest confidence comprised 34 genes including epigenetic regulators and adhesion molecules (Fig. 2A, B). All MSG1 MM displayed at least one hit within this core network, suggesting a common ground for the treatment of this subgroup. Indeed, 26 of the 241 MSG1 genes may be possible targets of FDA-approved drugs (Supplementary Table S8) and 10 of those (*PAPOLA* (poly(A) polymerase alpha), *PLAUR* (plasminogen activator, urokinase receptor), *COL18A1* (collagen type XVIII alpha 1 chain), *NR4A1* (nuclear receptor subfamily 4 group A member 1), *HDAC7* (histone deacetylase 7), *RB1* (retinoblastoma transcriptional corepressor 1), *COL1A1* (collagen type I alpha 1 chain), *EP300* (E1A-binding protein p300), *DDB1* (damage specific DNA-binding protein 1), *CHRNG* (cholinergic receptor nicotinic gamma subunit)) were comprised within the core signalling network of 34 genes and have been described in the context of cancer (Fig. 2B and Supplementary References). Most importantly, all but one MSG1 MM displayed a genetic hit in at least one of those ten drug-targetable genes (Fig. 2B).

**Fig. 2:**
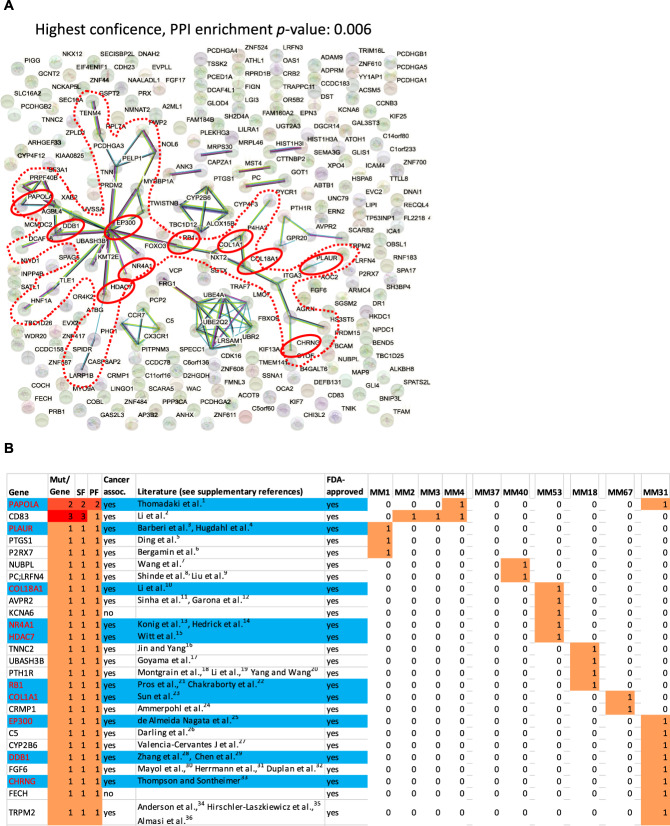
STRING analysis of MSG1 genes and the distribution of drug-associated MSG1 genes within the signalling network. The red dotted frame highlights the “core-network” of 34 genes and the red circles depict drug-associated genes—according to the Drug Gene Interaction Database (DGIdb)—within the core network (*n* = 10) (**A**). Distribution of drug-associated MSG1 genes (26/241) among MSG1 MM and their possible association with cancer according to the literature (for reference details, see Supplementary References). The blue colour highlights the 10 drug-associated core-network genes that were highlighted by red circles in Figure 2A (**B**). SF sample frequency, PF patient frequency, mut mutation.

In conclusion, all MM patients of the current cohort harboured mutations affecting RTK and adhesion signalling and, except for one patient, maintained their original MSG when further biopsies were analysed from later time points. This confirms the importance of the RTK and adhesion signalling network in myeloma^6^ and suggests that the genetic profiles defining MSG1 and MSG2 are robust and may play a central role in its pathogenesis. However, since only a fraction of patients was untreated at the time of sequencing and the study cohort was enriched for high-risk MM, it is difficult to determine whether RTK or RTK-effector mutations (which are restricted to MSG2 patients) are primary or secondary events. Nevertheless, the assignment of patients to either subgroup might improve risk stratification in addition to traditional molecular and clinical high-risk features^11,12^. In our study, despite the small sample size and heterogeneous treatment protocols, MSG1 patients had a favourable clinical course even in the presence of high-risk cytogenetic aberrations and may benefit from alternative or milder treatment approaches (Supplementary Fig. S2). In contrast, assessment of MYC expression and mutations of TP53, as well as RTKs and their effectors within the genomically more unstable MSG2, may allow to identify patients with a particularly dismal outcome who may benefit from the addition of specific drugs such as RTK- or PARP1-inhibitors to their treatment protocols.

Future investigations using a larger number of primary MM samples with known genetic profiles will further elucidate whether MSG1- and MSG2-assigned MM cells display different drug responses that may be translatable into the clinic.

## Supplementary information

S_Patients and Methods, S_Results and S_References

Figures S1 and S2

Tables S1, S2 and S7

Table S3

Table S4 A-C

Table S5 A-B

Table S6

Table S8
