# Supplementary material for: Novel molecular subgroups within the context of receptor tyrosine kinase and adhesion signalling in multiple myeloma
Source: Blood Cancer J. 2021 Mar 4;11(3):51. doi: 10.1038/s41408-021-00442-2 (PMC7933144; doi:10.1038/s41408-021-00442-2)
Supplement: Supplementary file 1 — S_Patients and Methods, S_Results and S_References [file 41408_2021_442_MOESM1_ESM.pdf]

Supplementary Patients and Methods

Multiple myeloma samples

Bone marrow aspirates (n=67) and corresponding peripheral blood mononuclear cells (PBMCs) were obtained from 43 MM patients (18 patients with longitudinal biopsies (1-4 samples per patient) treated at a single institution (Würzburg University Hospital) as part of their routine diagnostic workup after providing informed consent. Plasma cells were isolated using CD138 microbeads (Miltenyi Biotech, Bergisch Gladbach, Germany) as previously described[1] and DNA extracted using the AllPrep DNA/RNA mini or micro kits (Qiagen, Hilden, Germany). Only samples with a minimum DNA content of 100ng/µl, a 260/280 ratio of ≥ 1.8 and a 260/230 ratio of ≥ 1.9 were included in this study. Whenever possible, follow-up biopsies were obtained from patients on study for longitudinal monitoring.

Fluorescence in situ hybridization (FISH) for the detection of chromosomal gains in 1q21, 8q24, 9q34 and chr11, losses in 13q14 and 17p13 and the translocations (4;14), (14;16), (8;14) and (11;14) was performed in two certified laboratories according to standard protocols. With respect to sample selection for further genetic analyses we aimed for a good representation of high-risk MM, defined by the presence of a deletion of the short arm of chromosome 17 (del17p) in >60% of plasma cells and/or translocations t(14;16), t(14;20)[2,3], or extramedullary disease (EMD)[4]. The study was approved by the Ethics Committee of the Medical Faculty, University of Würzburg (reference number 18/09).

Whole exome sequencing

For preparation of primary MM and corresponding PBMC samples, the Paired-End DNA Sample Preparation Kit (Illumina, San Diego, CA, USA) was used. Amplification and exome enrichment were accomplished using the SureSelectXT Human All Exon kit (Agilent, Santa Clara, CA, USA). Sequences were generated with 2 x 100-125bp paired-end reads aiming at

a 90-fold mean coverage on target using the Illumina HiSeq-platform (Illumina, San Diego, CA, USA). Sequencing data are deposited at the European Genome-phenome Archive (EGA) under accession number EGAS00001003227.

PCR and Sanger sequencing

PCR and Sanger sequencing were performed using standard protocols. All primers (**Table S3**) were purchased from Integrated DNA Technologies (Leuven, Belgium).

Bioinformatic evaluation of sequencing data

Base calling, alignment, single nucleotide variant (SNV) calling and subsequent annotations were performed using CASAVA (configureBclToFastq.pl, Illumina), BWA (Burrows-Wheeler Alignment Tool), the HaploTypeCaller of GATK (Genome Analysis Toolkit) and SeattleSeq, as described previously[5]. To minimize the number of non-tumour-associated mutations, all SNPs/SNVs (listed in 1000 genomes and dsbSNPv130 and higher) were excluded from the datasets, and the remaining SNVs were filtered to comprise the missense, missense near splice site, stop-gained/lost, stop-gained/lost near splice site, splice-acceptor and splice-donor mutations. The visualization of gene-mutation frequencies and the screening of the generated SNV-dataset for mutations in adhesion molecules (n=642), RTKs (n=74) and their effectors (n=63) (**Table S1**) was accomplished with the help of Phyton-Scripts (“Genemapping” and “Heatmap”) and the integration of aliases/synonyms from the HGNC database. Moreover the “Genemapping” script allowed to match genes with name-analogy. Whether genes with name-analogy, that were not included in the initial list, could in fact be assigned to either RTKs, adhesion molecules or effectors was decided upon extensive literature research. STRING network analysis (STRING v11, https://string-db.org/) was used to identify a common signalling network among MSG1.

Immunohistochemistry

The MYC expression status was determined on deparaffinized formalin-fixed trephine biopsies using antibodies against MYC (clone Y69, rabbit, #ab32072 (Abcam, Cambridge, UK), citric acid ph 6.0) and CD138/Syndecan (clone MI15, mouse, #M7228 (Agilent, Santa Clara, CA, USA), citric acid ph 6.0) according to standard protocols. MYC expression in CD138-positive plasma cells was evaluated in 5% steps. According to recent findings[6], a case was assigned to be MYC^{high} when ≥40% of CD138-positive MM cells expressed MYC. Notably, ≥40% equals >30% in the current study cohort.

Statistical evaluation

For all correlations except for survival statistics, the Chi-square test was applied. In addition, the Mann-Whitney U test (MWU test) was applied for all correlations that included MYC expression and TMB. To factor the changes that can occur within one patient over time on the one hand and to avoid over-estimation of repetitive events within one patient on the other hand, the correlations were calculated at biopsy-level (including all biopsies of each patient) as well as on patient-level (the same patient was counted in the two different groups, if the respective information changed among the biopsies of the same patient, while patients with repetitive biopsies were counted only once). The Kaplan-Meier method was used to perform survival analyses and the log-rank test was used for group comparisons. *p*-values <0.05 were regarded as statistically significant. All statistical analyses were carried out and corresponding artwork was created using Prism software version 8.4 (GraphPad, La Jolla, CA, USA).

References

1. Stühmer T, Arts J, Chatterjee M, et al. Preclinical anti-myeloma activity of the novel HDAC-inhibitor JNJ-26481585. *Br J Haematol.* 2010;149(4):529-536.
2. Mikhael JR, Dingli D, Roy V, et al. Management of newly diagnosed symptomatic multiple myeloma: updated Mayo Stratification of Myeloma and Risk-Adapted Therapy (mSMART) consensus guidelines 2013. *Mayo Clin Proc.* 2013;88(4):360-376.

3. Pawlyn C, Morgan GJ. Evolutionary biology of high-risk multiple myeloma. *Nat Rev Cancer.* 2017;17(9):543-556.
4. Usmani SZ, Heuck C, Mitchell A, et al. Extramedullary disease portends poor prognosis in multiple myeloma and is over-represented in high-risk disease even in the era of novel agents. *Haematologica.* 2012;97(11):1761-1767.
5. Zamo A, Pischimarov J, Schlesner M, et al. Differences between BCL2-break positive and negative follicular lymphoma unraveled by whole-exome sequencing. *Leukemia.* 2018;32(3):685-693.
6. Moller HEH, Preiss BS, Pedersen P, et al. Myc protein overexpression is a feature of progression and adverse prognosis in multiple myeloma. *Eur J Haematol.* 2018.

Supplementary Results

Patient population

Clinical characteristics of the study population are shown in **Table S3**. Briefly, 11 patients (26%) had newly diagnosed MM (NDMM) and 32 (74%) were pretreated. The majority of patients had novel agents (PI and/or IMiDs) for induction and an autologous stem cell transplantation as first-line therapy and all patients received PI/IMiD-based treatment at first and subsequent relapses.

Treatment outcome did not differ significantly in patients treated with conventional chemotherapy-based regimens, thus all patients could be included in subsequent survival analysis. With a median follow-up of 48.8 months from study entry, 25 (58%) patients had died. Median OS was 84.3 months for the entire cohort and 24.4 months for patients with high-risk features (see definition in and the supplemental patients and methods section) as calculated from the time of diagnosis (**Figure S1 A,B)**. Because OS was very similar between the intermediate-risk and standard-risk groups (**Figure S1B**), and clearly distinct from the high-risk group (**Figure S1A,** *p*<0.001), we compared intermediate-/standard-risk vs high-risk MM in subsequent correlations that involved the risk-status.

Of note, one patient with a reported del17p (MM37-40), T/N (71/52; 85/86; 99/-;149/-) at relapse in 2010 was diagnosed with MM in 2006 (unknown risk-status) and survived for 13 years.

Sequencing output, quality control and technical validation

MM samples were sequenced on average with 64 521 143 reads (18 358 827-124 129 653), resulting in a mean coverage of 123x (55-218) after exclusion of PCR duplicates. The filtering strategy mentioned in material and methods revealed 3.821 SNVs, which were subjected to further analysis. According to the “Genemapping” approach, 18/67 MM samples from 12 patients were affected by SNVs in RTKs, 60/67 MM samples from 40 patients by

SNVs in adhesion molecules and 49/67 MM samples from 33 patients by SNVs in RTK effectors (**Table S5 A-D**). To assign MM samples to certain molecular categories (mutated in RTKs, adhesion molecules, effectors, *TP53*) at least one SNV/mutation had to be technically verified within this category by Sanger sequencing or at least three independent genes of this category had to be affected by a somatic mutation in this specific MM sample (**Table S4**, **Table S5 A-D**). In total, 275 selected SNVs were investigated by Sanger sequencing, of which 21 (8%) were disproven by Sanger sequencing and 19 (7%) could not be evaluated for technical reasons (**Table S4**). Retrospective assessment of clinical data revealed that according to multiparametric flow cytometry analysis the biopsy from patient MM11, treated with a PI/IMiD combination, contained only polyclonal plasma cells. For this reason, we excluded this sample from further analysis. Three longitudinal samples from another patient ((MM19-21), T/N (20/19; 97/-; 112/113)) also had to be excluded due to lack of clarity concerning the precise identity of these samples. In consequence, subsequent correlations were carried out on 63 biopsies from 41 patients (17 patients with longitudinal sequencing information).

Supplementary References

Cited in Figure 2B

1, 2, 3, 4, 5, 6, 7, 8, 9, 10 11, 12, 13, 14, 15, 16, 17, 18, 19, 20, 21 , 22 , 23, 24, 25, 26, 27, , 28, 29, 30, 31, 32, 33, 34, 35, 36

1. Thomadaki E, Havredaki M, Tsiapalis CM. PAP modulations in Daudi cells and Molt-3 cells treated with etoposide are mutually associated with morphological evidence of apoptosis. *Int J Biol Markers.* 2004;19(3):203-212.
2. Li Z, Ju X, Lee K, et al. CD83 is a new potential biomarker and therapeutic target for Hodgkin lymphoma. *Haematologica.* 2018;103(4):655-665.
3. Barberi S, Montagna G, Rossi L. Expression of urokinase plasminogen activator (uPA) in the leukocytes and tissues of patients with benign and malignant breast lesions. *Breast Dis.* 2019;38(1):15-23.
4. Hugdahl E, Bachmann IM, Schuster C, Ladstein RG, Akslen LA. Prognostic value of uPAR expression and angiogenesis in primary and metastatic melanoma. *PLoS One.* 2019;14(1):e0210399.
5. Ding L, Gu H, Lan Z, et al. Downregulation of cyclooxygenase1 stimulates mitochondrial apoptosis through the NFkappaB signaling pathway in colorectal cancer cells. *Oncol Rep.* 2019;41(1):559-569.
6. Bergamin LS, Capece M, Salaro E, et al. Role of the P2X7 receptor in in vitro and in vivo glioma tumor growth. *Oncotarget.* 2019;10(47):4840-4856.
7. Wang Y, Wu N, Sun D, et al. NUBPL, a novel metastasis-related gene, promotes colorectal carcinoma cell motility by inducing epithelial-mesenchymal transition. *Cancer Sci.* 2017;108(6):1169-1176.
8. Shinde A, Wilmanski T, Chen H, Teegarden D, Wendt MK. Pyruvate carboxylase supports the pulmonary tropism of metastatic breast cancer. *Breast Cancer Res.* 2018;20(1):76.
9. Liu Y, Chen X, Chen X, Yang X, Song Q, Wu H. High SALM3 Expression in Tumor Cells and Fibroblasts Is Correlated with Poor Prognosis in Gastric Cancer Patients. *Dis Markers.* 2019;2019:8282414.
10. Li J, Wang X, Zheng K, et al. The clinical significance of collagen family gene expression in esophageal squamous cell carcinoma. *PeerJ.* 2019;7:e7705.
11. Sinha S, Dwivedi N, Tao S, et al. Targeting the vasopressin type-2 receptor for renal cell carcinoma therapy. *Oncogene.* 2020;39(6):1231-1245.
12. Garona J, Sobol NT, Pifano M, et al. Preclinical Efficacy of [V4 Q5]dDAVP, a Second Generation Vasopressin Analog, on Metastatic Spread and Tumor-Associated Angiogenesis in Colorectal Cancer. *Cancer Res Treat.* 2019;51(2):438-450.
13. Konig SM, Rissler V, Terkelsen T, Lambrughi M, Papaleo E. Alterations of the interactome of Bcl-2 proteins in breast cancer at the transcriptional, mutational and structural level. *PLoS Comput Biol.* 2019;15(12):e1007485.
14. Hedrick E, Mohankumar K, Safe S. TGFbeta-Induced Lung Cancer Cell Migration Is NR4A1-Dependent. *Mol Cancer Res.* 2018;16(12):1991-2002.
15. Witt AE, Lee CW, Lee TI, et al. Identification of a cancer stem cell-specific function for the histone deacetylases, HDAC1 and HDAC7, in breast and ovarian cancer. *Oncogene.* 2017;36(12):1707-1720.
16. Jin Y, Yang Y. Bioinformatics-based discovery of PYGM and TNNC2 as potential biomarkers of head and neck squamous cell carcinoma. *Biosci Rep.* 2019;39(7).

17. Goyama S, Schibler J, Gasilina A, et al. UBASH3B/Sts-1-CBL axis regulates myeloid proliferation in human preleukemia induced by AML1-ETO. *Leukemia.* 2016;30(3):728-739.
18. Montgrain PR, Phun J, Vander Werff R, Quintana RA, Davani AJ, Hastings RH. Parathyroid-hormone-related protein signaling mechanisms in lung carcinoma growth inhibition. *Springerplus.* 2015;4:268.
19. Li S, Chen P, Zheng K, et al. beta-Alanine mediated inhibition of PTHR1suppresses the proliferation, invasion and tumorigenesis in metastatic human osteosarcoma U2OS cells. *Int J Biol Macromol.* 2018;111:1255-1263.
20. Yang Y, Wang B. PTH1R-CaSR Cross Talk: New Treatment Options for Breast Cancer Osteolytic Bone Metastases. *Int J Endocrinol.* 2018;2018:7120979.
21. Pros E, Saigi M, Alameda D, et al. Genome-wide profiling of non-smoking-related lung cancer cells reveals common RB1 rearrangements associated with histopathologic transformation in EGFR-mutant tumors. *Ann Oncol.* 2020;31(2):274-282.
22. Chakraborty G, Armenia J, Mazzu YZ, et al. Significance of BRCA2 and RB1 Co-loss in Aggressive Prostate Cancer Progression. *Clin Cancer Res.* 2020;26(8):2047-2064.
23. Sun S, Wang Y, Wu Y, et al. Identification of COL1A1 as an invasionrelated gene in malignant astrocytoma. *Int J Oncol.* 2018;53(6):2542-2554.
24. Ammerpohl O, Pratschke J, Schafmayer C, et al. Distinct DNA methylation patterns in cirrhotic liver and hepatocellular carcinoma. *Int J Cancer.* 2012;130(6):1319-1328.
25. de Almeida Nagata DE, Chiang EY, Jhunjhunwala S, et al. Regulation of Tumor-Associated Myeloid Cell Activity by CBP/EP300 Bromodomain Modulation of H3K27 Acetylation. *Cell Rep.* 2019;27(1):269-281 e264.
26. Darling VR, Hauke RJ, Tarantolo S, Agrawal DK. Immunological effects and therapeutic role of C5a in cancer. *Expert Rev Clin Immunol.* 2015;11(2):255-263.
27. Valencia-Cervantes J, Huerta-Yepez S, Aquino-Jarquin G, et al. Hypoxia increases chemoresistance in human medulloblastoma DAOY cells via hypoxiainducible factor 1alphamediated downregulation of the CYP2B6, CYP3A4 and CYP3A5 enzymes and inhibition of cell proliferation. *Oncol Rep.* 2019;41(1):178-190.
28. Zhang Y, Lei Y, Xu J, et al. Role of Damage DNA-Binding Protein 1 in Pancreatic Cancer Progression and Chemoresistance. *Cancers (Basel).* 2019;11(12).
29. Chen Z, Wang K, Hou C, et al. CRL4B(DCAF11) E3 ligase targets p21 for degradation to control cell cycle progression in human osteosarcoma cells. *Sci Rep.* 2017;7(1):1175.
30. Mayol G, Martin-Subero JI, Rios J, et al. DNA hypomethylation affects cancer-related biological functions and genes relevant in neuroblastoma pathogenesis. *PLoS One.* 2012;7(11):e48401.
31. Herrmann A, Haake A, Ammerpohl O, et al. Pipeline for large-scale microdroplet bisulfite PCR-based sequencing allows the tracking of hepitype evolution in tumors. *PLoS One.* 2011;6(7):e21332.
32. Duplan SM, Theoret Y, Kenigsberg RL. Antitumor activity of fibroblast growth factors (FGFs) for medulloblastoma may correlate with FGF receptor expression and tumor variant. *Clin Cancer Res.* 2002;8(1):246-257.
33. Thompson EG, Sontheimer H. Acetylcholine Receptor Activation as a Modulator of Glioblastoma Invasion. *Cells.* 2019;8(10).
34. Anderson KJ, Cormier RT, Scott PM. Role of ion channels in gastrointestinal cancer. *World J Gastroenterol.* 2019;25(38):5732-5772.
35. Hirschler-Laszkiewicz I, Chen SJ, Bao L, et al. The human ion channel TRPM2 modulates neuroblastoma cell survival and mitochondrial function through Pyk2, CREB, and MCU activation. *Am J Physiol Cell Physiol.* 2018;315(4):C571-C586.
36. Almasi S, Kennedy BE, El-Aghil M, et al. TRPM2 channel-mediated regulation of autophagy maintains mitochondrial function and promotes gastric cancer cell survival via the JNK-signaling pathway. *J Biol Chem.* 2018;293(10):3637-3650.
