# Supplementary material for: Novel molecular subgroups within the context of receptor tyrosine kinase and adhesion signalling in multiple myeloma
Source: Blood Cancer J. 2021 Mar 4;11(3):51. doi: 10.1038/s41408-021-00442-2 (PMC7933144; doi:10.1038/s41408-021-00442-2)
Supplement: Supplementary file 2 — Figures S1 and S2 [file 41408_2021_442_MOESM2_ESM.pdf]

Figure S1

A B

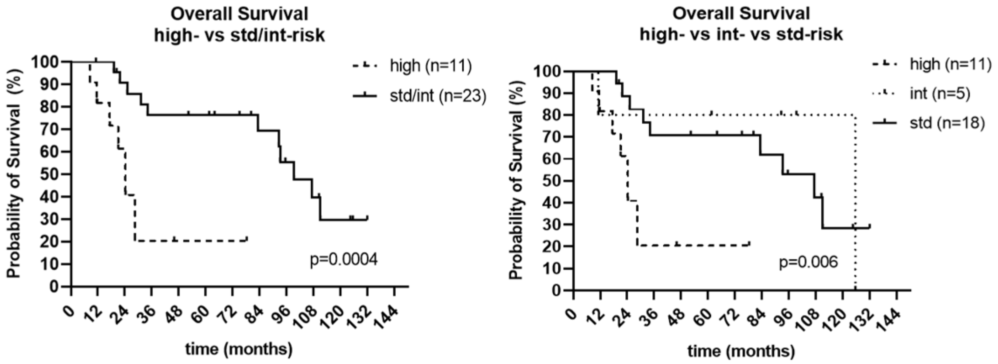

Figure S1 Correlation of risk-status with survival. Cases were either divided into two groups (high-risk vs intermediate/standard-risk) (**A**) or divided into three groups (high-risk vs intermediate-risk vs standard-risk) (**B**) according to the guidelines of the Mayo Clinic and the WHO (see material and methods).

Figure S2

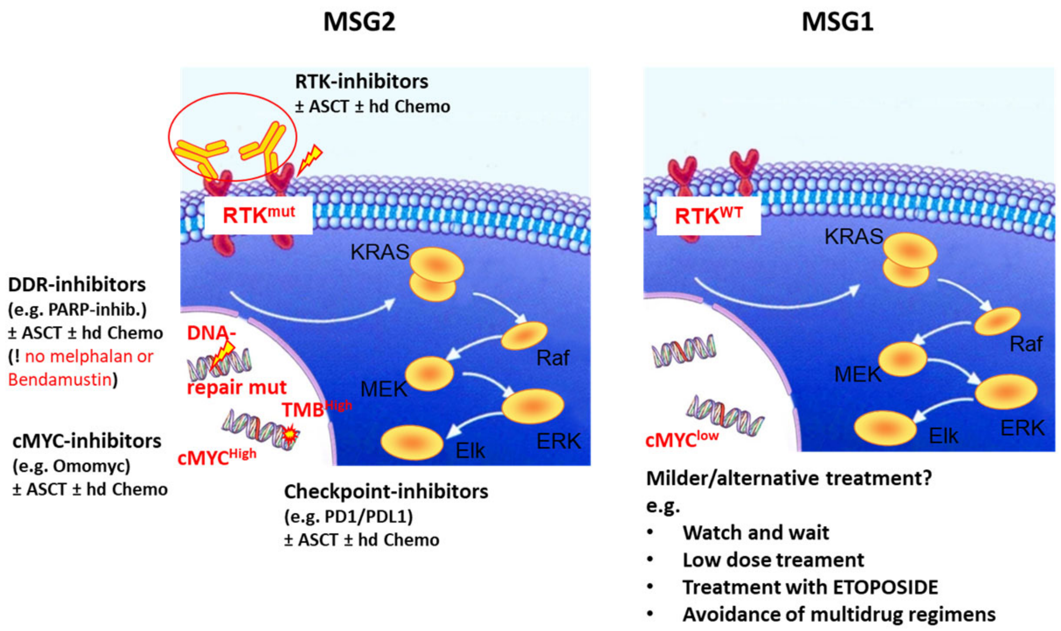

Figure S3 Hypothesis. MSG2-MM may require combined therapies, including for instance RTK- or PARP1-inhibitors while MSG-1 MM seems to constitute a more indolent subgroup that may profit from milder treatment options and the avoidance of multidrug regimens. DDR: DNA damage repair, ASCT: Autologous stem cell transplantation, hd: high dose, TMB: tumor mutational burden.
