# Supplementary material for: Novel molecular subgroups within the context of receptor tyrosine kinase and adhesion signalling in multiple myeloma
Source: Blood Cancer J. 2021 Mar 4;11(3):51. doi: 10.1038/s41408-021-00442-2 (PMC7933144; doi:10.1038/s41408-021-00442-2)
Supplement: Supplementary file 3 — Tables S1, S2 and S7 [file 41408_2021_442_MOESM3_ESM.pdf]

Table S1 A Genelist with RTKs used for the Phyton script "Genemapping"

BDNF
PDGFRA
PDGFRB
FGFR2
FGFR3
CSF1R
FLT4
FGR
TOM1L1
MUSK
NTRK2
IGF1R
EPHB2
EPHB1
NTRK3
NTF3
FYN
NTRK1
ERBB3
FGFR1
EPHA8
EGFR
EPHA2
DRL
DNT
EmRK2
ALK
TEK
AXL
EPHA5
CAD96Ca
KIT
CD117
MST1R
DDR1
TEK
INSR
FMS
MET
RET
SRC
DDR1
FGFR2
DDR2
SKY
EPHA1

EPHA3
EPHA4
EPHA5
EPHA7
EGFR2
ErbB2
ErbB4
FGFR1K
FGFR4
KDR
FLT3
GHR
EPHB3
EPHB4
JAK1
KGFR
LTK
EMR2
MET
TNK2
NGFR
ROS1
ROR2
RTK
CDK7
TIE1
RYK
VDR

Table S1 B Genelist with adhesion molecules used for the Phyton script "Genemapping"

CDH4
CDH6
CDH7
CDH8
CDH10
CDH11
CDH12
CDH13
CDH17
CDH20
CDH1
CDH15
CDH2
CDH3
CDH5
CDH9
CDH18
CDH22
CDH23
MUCDHL
DSC1
DSC2
DSC3
DSG1
DSG2
DSG3
DSG4
CELSR2
CELSR3
PCDH1
PCDH8
PCDH10
PCDHA1
PCDHA4
PCDH15
PCDHGC3
PCDHGA2
PCDHA2
PCDHA9
PCDHGA3
PCDHGB3
PCDHB6
ALCAM
AMICA
ASAM
BCAM

BOC
PECAM1
CDO
CEACAM1
CEACAM3
CEACAM4
CEACAM5
CEACAM6
CEACAM7
CEACAM8
L1CAM2
CLP24
CLP1
DSCAM
DSCAML1
EPCAM
ESAM
HEPACAM
HEPACAM2
ICAM1
ICAM2
ICAM3
ICAM4
ICAM5
IGSFA4
IGSF4B
IGSF4C
IGSF4D
IGSF2
IGSF11
IGSF3
IGSF22
IGSF8
IGSF1
ISLR2
JAMA
JAMB
JAMC
JAM4
NEGR1
NEPH1
NEPH3
NEPH2
LAMP
MADCAM1
MCAM
MDGA1
MDGA2

MFGE8
NCAM1
NCAML1
NCAM2
NPHS1
NFASC
NINJ1
NINJ2
NRCAM
OBCAM
OCAM
PEAR1
SALM2
SALM3
SALM4
SALM5
SIRPA
SIRPB
TCAM1
THBS1
THBS4
THSD1
TROP2
UBE2S
VCAM1
VSIG3
CLDN1
CLDN3
CLDN4
CLDN6
CLDN8
CLDN10
CLDN11
CLDN12
CLDN17
CLDN19
CNTN1
CNTN2
CNTN3
CNTN4
CNTN5
CNTN6
CNTNAP2
AGRN
TGFBI
LYPD3
CHI3L1
CHI3L2

ECFL
CHIA
CILP1
COCH
COL1
COL1A1
COL2
COL4
COL4A1
COL3A1
COL13A1
COL23A1
COL25A1
COL22A1
COL7A1
COL17A1
COL6A3
COL10A1
COL22A1
COLEC10
COLEC12
COL6A2
COL24A1
COL9A2
COL9A1
COL8A1
COL4A5
COL4A1
COL19A1
COL14A1
COL6A6
COL5A1
COL11A1
COL4A4
COL18A1
COL16A1
COL12A1
COMP
CRISP3
CRTAC1
CCN2
CTHRC1
CCN1
DPT
DMP1
ECM1
COL18A1
SPON1

FMOD
FN1
FBLN1
DANCE
FLRT1
FLRT2
FLRT3
IL32
IL32A
IL32B
IL32G
LAMA1
LAMA3
LAMA4
LAMC1
LAMB2
LAMA1
LAMA2
LAMB2
LAMB3
LAMA5
LAMC2
LAMB1
MFAP2
MFAP5
MATN2
MATN3
MATN4
MIA
SPON2
NPNT
NID1
NID2
CCN3
OPTC
PRELP
FREM1
RSPO2
RELN
SMOC1
SMOC2
SOD3
SPARC
SPARCL1
EGFL7
TNC
TNR
THBS1

THBS2
THBS4
TINAG
TGM2
TGM3
TGM4
TGM7
TGM6
VTN
VWF
WARP
CCN4
ITGA1
ITGA1B1
ITGA11B1
ITGA2
ITGA2B1
ITGA2B
ITGA3
ITGA3B
ITGA4
ITGA4B1
ITGA4B7
ITGA5
ITGA5B1
ITGA6
ITGA6B4
ITGA7
ITGA8
ITGA9
ITGA9B1
ITGAE
ITGAEB7
ITGAL
PARVA
TGBB1
TGB2
TGFBR3
TGFBR2
CD47
LAMP3
CD151
CYTH1
DMP1
EDIL3
EGFL6
IBSP
ICAP1

ILK
ITGB1BP2
MEPE
MFGE8
ITGALB2
ITGAM
ITGAMB2
ITGAV
ITGAVB1
ITGAVB3
ITGAVB5
ITGAVB6
ITGAVB8
ITGAX
ITGAXB2
ITGA10
ITGA10B1
ITGA11
ITGB1
ITGB2
ITGB3
ITGB4
ITGB5
ITGB6
ITGB7
ITGB8
MIG2
MEDD9
NID1
NID2
NIF
OPN
PXN
PINCH1
RAGE
APBB1IP
TLN1
TLN2
TNXB
TINAG
TSPAN8
TSPAN9
TSPAN1
TSPAN7
ASGPR1
/CD93
FCER2
CD72

KLRD1
CLEC13A
CHODL
COLEC11
COLEC12
COLEC10
CLEC1
CLEC2
CLEC3B
CLEC4D
CLEC4F
CLEC9A
CLEC2A
CLEC10A
CLEC14A
CLEC4L
DCSIGN
DCSIGNR
CLEC4B
CLEC4A
DEC205
CLEC7A
CLEC6A
CLEC6A
CLEC12B
FCN1
FCN2
FCN3
KLRE1
CD207
LAYN
LOX1
CLEC4G
MBL
MBL1
MBL2
CLEC5A
MGL1
MGL2
CLEC12A
MMR
MRC2
NKG2A
NKG2C
NKG2D
NKG2H
KLRF1
CLEC2d

CLEC2I
PLA2R1
FREM1
REG1
REG1A
REG1B
REG2
REG3A
REG3B
REG3D
REG4
CLEC11A
SIGNR1
SIGNR3
SIGNR4
SIGNR7
SPD
LGALS1
LGALS2
LGALS3
MAC2BP
LGALS4
LGALS7
LGALS8
LGALS9
LGALS10
LGALS14
CD83
SIGLEC4A
SIGLEC1
SIGLEC2
SIGLEC3
SIGLEC5
SIGLEC6
SIGLEC7
SIGLEC9
SIGLEC10
SIGLEC11
SIGLEC14
SIGLEC16
SIGLECE
SIGLECF
SELE
SELL
SELP
ITLN1
LMAN1L
PVRL1

PVRL2
PVRL3
PVRL4
CD44
HABP1
HAPLN1
HAPLN4
HAS1
HASL
HYAL4
HYAL3
LAYN
LYVE1
NCAN
SPAM1
STAB1
STAB2
TSG6
ACAN
AGRN
BGN
BCAN
DCN
DSPG3
DAG1
ESM1
PODLX2
HSPG2
FMOD
GPC1
GPC2
GPC3
GPC5
GPC6
LUM
OGN
ARSA
ARSB
ARSG
ARSD
ARSE
GUSB
CHST15
EXTL1
EXTL2
EXTL3
GNS
NCAN

CSPG5
NG2
OPTC
OSAD
PODN
PRELP
SGCD
SDC1
SDC2
SDC3
SDC4
SPOCK1
SPOCK2
SPOCK3
VCAN
HS2ST1
HS3ST1
HS3ST4
HS6ST1
HS6ST2
HS6ST3
IDS
SULF2
WARP
AMIGO
AMIGO2
AMIGO3
MUC16
MUC21
MUC4
MUC20
MUC6
MUC17
MUC13
MUC12
MUC2
MUC5AC
MUC7
CD2
CD6
CD9
CD34
SRB3
CD43
CD44
LFA3
CD63
SLAMF5

CD90
CD96
CD98
CD99
CD99L2
CD164
SLAMF3
DOCK3
DOCK1
DOCK7
GPR56
GPR101
GPR112
GPRASP1
GPR124
GPR155
GPR142
GPR199
GPR64
GPR111
GPR45
GPR123
GPR116
GPR125
GPR133
GPR156
GPR158
GPRC6A
GPR114
GPR78
GPRC5B
GPR115
GPR110
GPR113
HIC5
MUC1
NRXN1A
NXRN1B
NRXN2A
NRXN3
NRXN3B
NRXN1
ODZ1
ODZ2
ODZ3
ODZ4
VCL
ADAM8

ADAM9
ADAM10
ADAM12
ADAM19
ADAMTS1
ADAMTS3
ADAMTS4
ADAMTS5
ADAMTS8
ENPEP
ERAP2
ANPEP
ONPEP
XPNPEP1
XPNPEP2
ARTS1
DPEP3
DPEP1
ANPEP
FNPEP
RNPEPL1
CPA1
CPA2
CPA4
CPB1
MMP1
MMP2
MMP3
MMP7
MMP8
MMP9
MMP10
MMP11
ADAM22
ADAM23
ADAM32
ADAM33
ADAM17
ADAMTS10
ADAMTS13
ADAMTS15
ADAMTSL1
METAP
METAP1
METAP1D
METAP2
CPB2
CPE

CPXM1
PRCP
CPGL
MMP12
MMP13
MMP14
MMP15
MMP16
MMP19
MMP24
MMP25
SPOCK1
SPOCK2
SPOCK3
TIMP
TIMP1
TIMP2
TIMP3
TIMP4
PLAUR

Table S1 C Genelist with effector molecules used for the Phyton script "Genemapping"

PLCG
DAG
IKK
PKC
cAMP
AKAP
PKA
PKAC
GRK2
SOS
GBR2
SHC
GAB1
IRS
JAK
RAS
RAF
MEK
ERK
STAM
PI3K
PIP2
PIP3
PDK1
AKT
MTOR
BAD
TYK2
STAT
IRF9
PTEN
HK3
GSK
FOXO
FYN
SHC
GRB2
SOS
FAK
SRC
RHOGAP
ROCK
MLC
CDC42
PAK

JNK
CASP
DAB1
MAPK
PI3K
ERK
ILK
AKT
FRAP
PXN
VCL
VASP
RAS
RAF
MEK
MAPK
ERK
PTK2B

Table S2: Clinical characteristics of the study population

Characteristic	Value (n = 43)
**Age, median (range), yr**	66.2 (49.9-87.1)
**Male sex, n (%)**	27 (63)
**Type of myeloma, n (%)**	
IgG	25 (58)
IgA	13 (30)
Light chain	5 (12)
**BM plasma cell infiltration, (%)**	
<30%	7 (16)
30-59%	12 (28)
≥60	23 (54)
Data not available	1 (2)
**Cytogenetic abnormality, n (%)**	
**del17p**	
Yes	10 (23)
No	27 (63)
Data not available	6 (14)
**t(4;14)**	
Yes	10 (23)
No	26 (61)
Data not available	7 (16)
**t(14;16)**	
Yes	2 (5)
No	31 (72)
Data not available	10 (23)
**amp1q21**	
Yes	14 (33)
No	20 (46)
Data not available	9 (21)
**hyperdiploid**	
Yes	7 (16)
No	24 (56)
Data not available	12 (28)
**High-risk disease at study entry, n (%)**	
Yes	15 (35)
Data not available	7 (16)
**Treatment-naïve patients, n (%)**	11 (26)
**Median No. of prior treatment regimens in pretreated pts (range)**	1 (1–8)
**Median time since initial diagnosis, yr (range)**	1.7 (0-9.2)
**Eligible for autologous stem cell transplantation, n (%)**	33 (77)
**Novel agents for induction, n (%)**	34 (79)

Table S7 Comparisons and their corresponding *p*-values

**Correlation/Comparison**	**Figure**	**Fisher's Exact (patient-level)**	**Fisher's Exact (biopsy-level)**	**MWU (biopsy-level)**	**log-rank**
MSG1 vs MSG2 (MYC-expr.)	Fig. 1C,D	$p<0.05$	$p<0.01$	$p<0.01$	-
MYC^{hig} vs MYC^{low} (OS)	Fig. 1E	-	-	-	$p=0.028$
High vs interm./low-risk (RKT mut-status)	-	$p=0.245$	$p=0.324$	-	-
RTK^{mut} vs RTK^{WT} (OS)	-	-	-	-	$p=0.448$
High vs interm./low-risk (MYC expr-status)	-	$p=0.041$	$p=0.0767$		-
RTK^{mut} vs RTK^{WT} (MYC expr.)	-	$p=0.147$	$p=0.183$	-	-
High vs interm./low-risk (MYC expr.-/RKT mut-status)	Fig. 1G	$p<0.01$	$p=0.04$	-	-
MYC^{high} vs MYC^{low} (OS)	Fig. 1H	-	-	-	$p=0.021$
MSG1 vs MSG2 (TMB)	-	-	-	$p=0.012$	-
TP53-dh vs TP53-sh (OS)	-	-	-	-	$p=0.105$
